# Multi-activity cobalt ferrite/MXene nanoenzymes for drug-free phototherapy in bacterial infection treatment

**DOI:** 10.1039/d2ra01133f

**Published:** 2022-04-08

**Authors:** Jiacheng Shi, Rui Shu, Xiuyuan Shi, Yunfei Li, Jiangge Li, Yi Deng, Weizhong Yang

**Affiliations:** College of Biomedical Engineering, School of Chemical Engineering, Sichuan University Chengdu 610065 China dengyibandeng@scu.edu.cn ywz@scu.edu.cn; State Key Laboratory of Oral Diseases, National Clinical Research Centre for Oral Disease, Department of Orthodontics and Pediatrics, West China Hospital of Stomatology, Sichuan University Chengdu Sichuan 610041 China; State Key Laboratory of Polymer Materials Engineering, Sichuan University Chengdu 610065 China; Department of Materials, Imperial College London SW7 2AZ London UK; Department of Biomedical Engineering, The City College of the City University of New York New York USA

## Abstract

Drug-free antibacterial strategies are of great significance for pathogenic bacterial infection treatment in clinical practice. Phototherapy with antibacterial function plays a vital role in mainstream germicidal research. However, phototherapy could lead to residual heat and excess reactive oxygen species (ROS), which are the main side-effects during antibacterial treatment. Unique CoFe_2_O_4_/MXene (CM) nanoenzymes, which were fabricated with electrostatic interactions, have been designed to conquer those challenges caused by side-effects of phototherapy in our research. The CM nanoenzymes possess many promising properties including photothermal and photodynamic induced phototherapy and mimic peroxidase (POD), glutathione oxidase (GSHOx), and catalase (CAT). Upon treatment with near-infrared (NIR) light, CM nanoenzymes can create a local high-temperature circumstance as well as raise bacterial membrane permeability. Furthermore, the photodynamic process and multi-enzyme-mimicking activities of CM enzymes boost the interbacterial ROS level. Herein, bacteria can hardly survive in synergistic phototherapy and multi-enzyme-mimicking catalytic therapy *in vitro* and *in vivo*. Meanwhile, the CM nanoenzymes exhibit excellent biocompatibility *in vitro* and *in vivo*. Overall, this research establishes a strong foundation for effectively employing nanoenzymes, leading to a new way to cure bacterial infections.

## Abstract 2

The CM nanoenzymes are synthesized with electrostatic interactions. Bacteria can hardly survive synergistic phototherapy and multi-enzyme-mimicking catalytic therapy with CM, which reinforces the foundation of drug-free antibacterial strategies.

## Graphical abstract

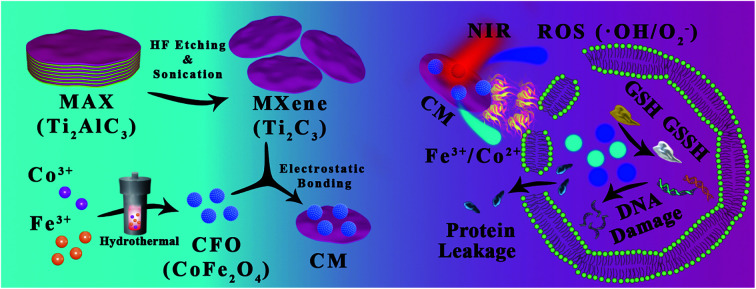

## 1. Introduction

Despite the advancement in medicine and pharmaceutics, pathogenic bacterial infection remains one of the most severe hazards to people's health around the world.^1,2^ Specifically, significant microbial infection and long-term inflammation would result in implant-associated disease or chronic stalled wounds.^3,4^ Nowadays, the standard treatment for bacterial infection is systemic antibiotics.^5^ However, with the abuse and misuse of antibiotics, bacteria have been shown to be drug-resistant and evolved to be multidrug-resistant.^6^ Furthermore, bacterial resistance has necessitated a continuing increase in antibiotic use, resulting in widespread environmental degradation.^7^ In terms of public health safety and financial losses, bacterial infection would cause an onerous burden in the future.^8^

With the limitations of traditional antibiotics, antibiotics-free antibacterial strategies have been explored to kill bacteria.^9,10^ There are two main kinds of sterilization mechanisms without antibiotics.^11^ One is metallic ions therapy, which utilizes bactericidal metal ions (such as Ag and Zn ions) and has been used widely around the world in decades.^12,13^ Ag nanoparticles have become one of the most exciting marketable nanomaterials in the biomedical area because of their well-known sterilization activity.^14,15^ The positively charged metallic ions contact the negatively charged membrane of bacteria by electrostatic interaction.^16^ The antibacterial effects severely depend on the concentration of released ions.^17^ However, those ions result in cytotoxicity at high concentrations.^18^ Another mechanism for sterilization without antibiotics is phototherapy, which focuses on the light-triggered bactericidal process.^19,20^ Local hyperthermia-related photothermal therapy (PTT) and reactive oxygen species (ROS), containing superoxide radical (˙O_2_^−^), hydroxyl radicals (˙OH), and singlet oxygen (^1^O_2_), relevant photodynamic therapy (PDT) are two primary types of phototherapies. PTT depends on photothermal transfer agents for the generation of hyperthermia in a particular area to kill bacteria.^21^ PDT relies on photocatalyst to produce ROS for a breakdown in bacterial metabolism.^22^ Nevertheless, when the temperature reaches as high as 60 °C, local hyperthermia would cause irreversible damage for the normal tissue with a long-time effect.^23^ ROS generated due to irradiation hardly enters bacteria by a protection of the complete membrane structure.^24,25^ Therefore, a novel antibacterial model should be designed and applied with the combination of the original basis.

It is reported that bacterial infectious microenvironment (IME) has low pH (about 5.0) and high levels of H_2_O_2_, which is of benefit to Fenton or Fenton-like reaction.^26–28^ Fe(ii) or Co(ii) catalyze H_2_O_2_ to produce ˙OH effectively through Fenton or Fenton-like reaction.^29,30^ Fe(iii) and Co(iii) can oxidize glutathione (GSH) to GSSG and produce Fe(ii) or Co(ii) as reduced products.^31^ Fe(iii)/Fe(ii) and Co(iii)/Co(ii) exhibit peroxidase-mimicking (POD-mimicking) and glutathione oxidase-mimicking (GSHOx-mimicking) activity in the redox cycle.^32,33^ Previous research demonstrated that POD-mimicking and GSHOx-mimicking properties can improve the antibacterial effect by significantly declining GSH level and dramatically elevating ROS level.^34^ Besides, the catalase-mimicking (CAT-mimicking) activity of Fe(iii) can catalyze H_2_O_2_ to produce oxygen (O_2_), which greatly ameliorates the hypoxia state in IME.^35,36^ The oxygen-rich condition can improve the ROS generation from the PDT process with near-infrared (NIR) light and promote angiogenesis and tissue regeneration in the dark.

Cobalt ferrite (CoFe_2_O_4_, CFO) contains multivalent Co and Fe ions, which possesses favorable NIR light absorption, photocatalytic property, and excellent various enzyme-mimicking catalytic activities.^37–39^ Moreover, Fe and Co ions are favorable for tissue regeneration and angiogenesis.^40,41^ A viable technique for creating and producing light-responsive antibacterial agents with higher effectiveness combines and interacts well with nanoparticles and two-dimensional (2D) nanomaterials.^34^ Due to its 2D planar structure, semiconductor uniqueness, and enthralling photoelectric capabilities, the emerging 2D material Ti_3_C_2_ MXene has attracted a lot of attention.^42–44^ It has been reported to be applied in the scope of antibacterial agents due to aforesaid benefits.^45^ In order to combine multivalent Co and Fe ions on MXene, CFO was chosen to be applied in synthesis of nanoenzymes CFO/MXene (CM). CM nanoenzymes are endowed with phototherapy and enzyme-mimicking activities. When NIR is applied, CM nanoenzymes generate a local high thermal environment to gradually disintegrate bacterial membrane structure.^46^ The increase of bacterial membrane permeability facilitates that ROS generated in the photodynamic process access into bacteria.^47^ Meanwhile, it is convenient for photo-induced Co and Fe ions that disengage from CFO nanoparticles to execute multi-enzyme-mimicking catalysis within bacteria. These effects will disorder the self-protective mechanism of bacteria, which may lead to the superior antibacterial potency. While NIR is withdrawn, nanoenzymes can take a limited antibacterial effect and encourage tissue regeneration.

Given these previous considerations, the importance of the present research is to develop CM nanoenzymes combining CoFe_2_O_4_ and Ti_3_C_2_ MXene for antibacterial therapy with a synergy of multi-enzyme-mimicking catalytic activity, NIR-induced photothermal and photodynamic effect. Although some existed studies concerned MXene and other nanomaterials serving as antibacterial agents, the dependence on phototherapy leaded to potential harms for normal tissues without further treatment. The bactericidal effects of CM nanoenzymes against *Staphylococcus aureus* (*S. aureus*, Gram-positive pathogenic bacteria) and *Escherichia coli* (*E. coli*, Gram-negative pathogenic bacteria) were detected in the designed experiment. Furthermore, the mechanism of antibacterial effect about CM nanoenzymes was scrutinized at the same time in this research.

## 2. Experimental section

### 2.1. Synthesis of CFO/MXene (CM) nanoenzymes

MXene (Ti_3_C_2_) was synthesized by *in situ* HF etching of the MAX (Ti_3_AlC_2_, 11 Technology, Jilin, China). 1 g LiF (Aladdin, Shanghai, China) was added into 20 mL, 9 mol mL^−1^ HCl (Chengdu Kelong, Sichuan, China) under stirring in an ice bath. Then 1 g MAX was added into the mixture at 35 °C for 24 h. CFO was synthesized with the hydrothermal method. 0.36 g FeCl_3_·6H_2_O (Aladdin), 0.38 g CoCl_2_·6H_2_O (Aladdin), 1.8 g sodium acetate (Aladdin) and 0.5 g NaOH (Aladdin) were added into 40 mL ethylene glycol solution. The mixture was transferred into Teflon autoclave and kept at 180 °C for 0.5 d. Finally, the CFO powder was collected, rinsed by ethyl-alcohol and deionized (DI) water before drying. CM was synthesized *via* the sonication method. 5 mg MXene and 5 mg CFO were dissolved in 10 mL DI water and sonicated for 0.5 h in the ice bath.

### 2.2. Materials characterization

Scanning electron microscope (SEM, JSM-7500-F, JEOL, Japan) equipped with energy dispersive spectrometer (EDS) mapping and transmission electron microscope (TEM, Tecnai G2 F20 S-TWIN, FEI, USA) were used for detecting the surface morphologies and microstructures of samples. The crystal structure of materials was characterized by X-ray diffraction (XRD, Xcalibur A Ultra, Oxford, UK). The potentiometric analyzer (Zetasizer Nano-ZS-90, Malvern, UK) was utilized to figure out zeta potential of different samples.

### 2.3. Photothermal performance

To determine the photothermal property of materials, 808 nm NIR light was employed to irradiate samples, and the thermal infrared imager (FLIR, E6, USA) was employed to track the temperature variation. Samples were dispersed in phosphate-buffered solution (PBS). 0.5 mL of suspension in different concentrations (0.5, 1.0, and 2.0 mg mL^−1^) was exposed under the NIR light (0.5, 1.0, and 1.5 W cm^−2^) for 10 min, and the temperature was recorded every minute. Furthermore, the photothermal stability was also conducted for three heating and cooling cycles.

### 2.4. Photodynamic performance

1,3-Diphenylisobenzofuran (DPBF, Aladdin) was used as the O_2_^−^ trapping agent to detect the O_2_^−^ generation from samples. A mixture of DPBF and sample was solved and irradiated under NIR light for 10 min. Afterwards, UV-vis spectrophotometer (UV-1800 PC, AOELAB, China) was used to investigate the absorbance.

The existence of ˙OH was detected using methylene blue (MB, Aladdin) and rhodamine B (RhB) as ˙OH trapping agents. A mixture of MB/RhB and sample was solved and irradiated with NIR for 10 min. UV-vis spectrophotometer was employed to measure the absorbance of the solution.

### 2.5. Antibacterial activity

*E. coli* (ATCC 25922) and *S. aureus* (ATCC 25923) were used as typical Gram-negative and Gram-positive bacteria. A mixture of 0.5 mL bacterial suspensions (5.0 × 10^4^ CFU mL^−1^) and 0.5 mL sample (1.0 mg mL^−1^) was irradiated with 1.5 W cm^−2^ NIR light for 10 min or kept in dark as control. Treated mixture was seeded on Luria–Bertani (LB) agar plates by spread plate method. Take count of the number of bacteria colonies after overnight incubation at 37 °C. The bactericidal rate (BR) was computed as eqn (1):

1 BR% = (*C*_CFO_ − *C*_Sa_)/*C*_CFO_ × 100%

where *C*_CFO_ is the number of bacteria of the CFO sample in the dark and *C*_Sa_ is the number of bacteria in other samples.

The bacteria treated with different manners were incubated with LIVE/DEAD™ BacLight™ Bacterial Viability Kit (Thermo Fisher, USA) and captured by inverted fluorescent microscope (DMi 8, Leica Microsystems, Germany). The quantitative analysis of fluorescent intensity was performed by ImageJ.

### 2.6. Bacterial morphologies observation

The morphology of bacteria was observed by SEM to further determine the antibacterial properties of samples. The bacteria suspension was incubated on the cell-attached slide for 4 h and treated in the same way as described before in 2.5. Afterwards, the slides were fixed with 2.5% paraformaldehyde and dehydrated using gradient ethanol series (30, 50, 60, 70, 80, 90, and 100%) for SEM detection.

### 2.7. Bacterial membrane permeability test

*O*-Nitrophenyl-β-d-1-thiogalactopyranoside (ONPG, Aladdin) assay was used to achieve the permeability assay of the bacterial membrane. *E. coli* and *S. aureus* (10^7^ CFU mL^−1^) were cocultured with 10 μg mL^−1^ isopropyl-β-d-thiogalactopyranoside (IPTG, Aladdin) at 37 °C overnight. Then the bacteria were treated as before in 2.5. 20 mM ONPG was then added into the mixture. Microplate reader (SAF-6801, BAJIU, China) was employed to identify the absorbance of the supernatant at 410 nm.

### 2.8. ROS level in bacterial

The ROS in bacteria were detected by 2,7-dichlorodihydrofluorescein diacetate (DCFH-DA, Solarbio). The bacterial suspension was co-cultured with DCFH-DA 30 min for probe loading and treated as described before in 2.5. The images were captured by fluorescence microscope (CKX53, OLYMPUS, Japan).

### 2.9. GSH oxidase-mimicking activity

Different samples were mixed with GSH (0.8 mM) in carbonate buffer (pH = 9.6) and treated with the 1.5 W cm^−2^ NIR light for 10 min or kept in dark. Afterwards, Tris buffer (pH = 8.0) and DTNB (10 mM) were added into the mixture. The absorbance value of the solution was detected at 410 nm. H_2_O_2_ were set as positive control and PBS were set as negative control. The GSH consumption rate was computed as eqn (2):

2 GSH consumption rate = (OD_Ne_ − OD_Sa_)/(OD_Ne_ − OD_Po_) × 100%

where OD_Ne_, OD_Po_, and OD_Sa_ are the optical density (OD) value at 410 nm of negative control groups, positive control groups, and samples separately.

### 2.10. Peroxidase-mimicking property

Peroxidase can catalyse the oxidation of a substrate by H_2_O_2_ and produce ox-produce. TMB solution (2 mM) was mixed with H_2_O_2_ (3 mM) and samples. 10 min later, acetate buffer was added into the mixture. The absorbance of the solution was detected by the UV-vis spectrophotometer.

### 2.11. CAT-mimicking activity

H_2_O_2_ (3 mM) was added into different samples solutions. The oxygen content was detected by a portable dissolved oxygen meter (JPB-607A, INESA, China).

### 2.12. Hemocompatibility test

Hemolysis experiment was carried out to ensure that the samples were hemocompatible. DI water was set as positive control and PBS were set as negative control. Whole blood of goat was diluted 1 : 50 with PBS to prepare erythrocyte solution. Samples were cocultured with erythrocyte solution at 37 °C for 3 h. Furthermore, the mixture was centrifuged at 4 °C, and the absorbance at 545 nm of supernatant was detected. The hemolysis rate was computed as eqn (3):

3 Hemolysis rate = (OD_Sa_ − OD_Ne_)/(OD_Po_ − OD_Ne_) × 100%

where OD_Sa_ is the OD values of different samples. OD_Ne_, and OD_Po_ are OD values of negative control groups, and positive control groups, separately.

The effect of materials on coagulation ability reflects in the impact on activated partial thromboplastin time (APTT), prothrombin time (PT), thrombin time (TT), and fibrinogen (FIB). Blood cells were removed from the whole blood of the sheep by centrifugation and plasma was collected. The plasma was mixed with samples. Pure plasma was set as control. Coagulation analyzer (PUN-2048B) was used to conduct coagulation tests.

### 2.13. Cytotoxicity evaluation

L929 mouse fibroblasts were maintained and expanded in high-glucose DMEM (Gibco, USA) supplemented with penicillin (100 U mL^−1^), streptomycin (100 μg mL^−1^) and 10% fetal bovine serum (FBS, Gibco, USA), at 37 °C in 5% CO_2_ atmosphere. 1.0 × 10^4^ L929 cells were seeded in the cell of 48-well plates. After 1 day-incubation, samples dispersed in DMEM (0.5, 1.0 and 2.0 mg mL^−1^) were added and incubated for 1, 3, and 5 d. Cytotoxicity evaluation was achieved by Cell Counting Kit-8 (CCK-8) assay, and OD values at 450 nm were detected by microplate reader. The viability of L929 cells incubated with different samples (1.0 mg mL^−1^) after irradiated with 1.5 W cm^−2^ NIR light for 10 min, was also evaluated. After incubation 3 d with different samples, L929 cells were stained with FITC-phalloidin (Solarbio, China) and 4′,6-diamidino-2-phenylindole (Solarbio, China). Finally, samples were washed with PBS and captured by an inverted fluorescence microscope.

### 2.14. *In vivo* evaluation

The Animal Care and Experiment Committee of West China Hospital of Sichuan University (2018045A) approved all animal experiment procedures in this study, which followed the NIH Guide for the Care and Use of Laboratory Animals recommendations. Female BALB/c mice (6–8 weeks, Byrness Weil Biotech Ltd) were anesthetized with 10% chloral hydrate, the hair on back of the mice was removed and a full thickness wound with a diameter of 6 mm was made on the back of each mouse by surgical scissors. 10 μL of 2 × 10^8^ CFU mL^−1^*S. aureus* was added onto the wound and bandaged with airtight dressing. After 24 h the infectious wound model was constructed.

The mice were randomly divided into 7 groups with different treatments as: PBS, CFO NIR^−^, MXene NIR^−^, CM NIR^−^, CFO NIR^+^, MXene NIR^+^, and MX NIR^+^ (*n* = 3). Different groups were treated with 50 μL samples (1.0 mg mL^−1^). NIR^+^ groups were irradiated with 1.5 W cm^−2^ NIR light for 10 min. After the treatment, the wound exudates of each group were collected in 1 mL LB agent to spread on LB agar plate. The collected wound exudates were incubated for 24 h at 37 °C and measured at 600 nm. The *in vivo* BR was computed as eqn (4):

4 *In vivo* BR% = (OD_T_ − OD_L_)/(OD_P_ − OD_L_) × 100%

where OD_T_ is the OD values of different samples. OD_P_, and OD_L_ are OD values of PBS groups, and LB agent separately.

The hearts, livers, spleens, lungs, and kidneys were harvested from sacrificed mice on 5 d. Biocompatibility and biosafety of materials were detected by the H&E stain of organs.

### 2.15. Statistical analysis

Quantitative data are expressed as mean values ± standard deviation (SD). One-way analysis of variance (ANOVA) was performed to compared between numerous groups, followed by Tukey's multiple comparison test, which was conducted by GraphPad Prism 9.0 software (Graphpad Software, Inc., La Jolla, USA). Value of *p < 0.05 was reckoned to be significant. All tests were performed in at least 3 independent replicates.

## 3. Results and discussion

### 3.1. Synthesis and characterization of CM nanoenzymes

**Fig. 1:**
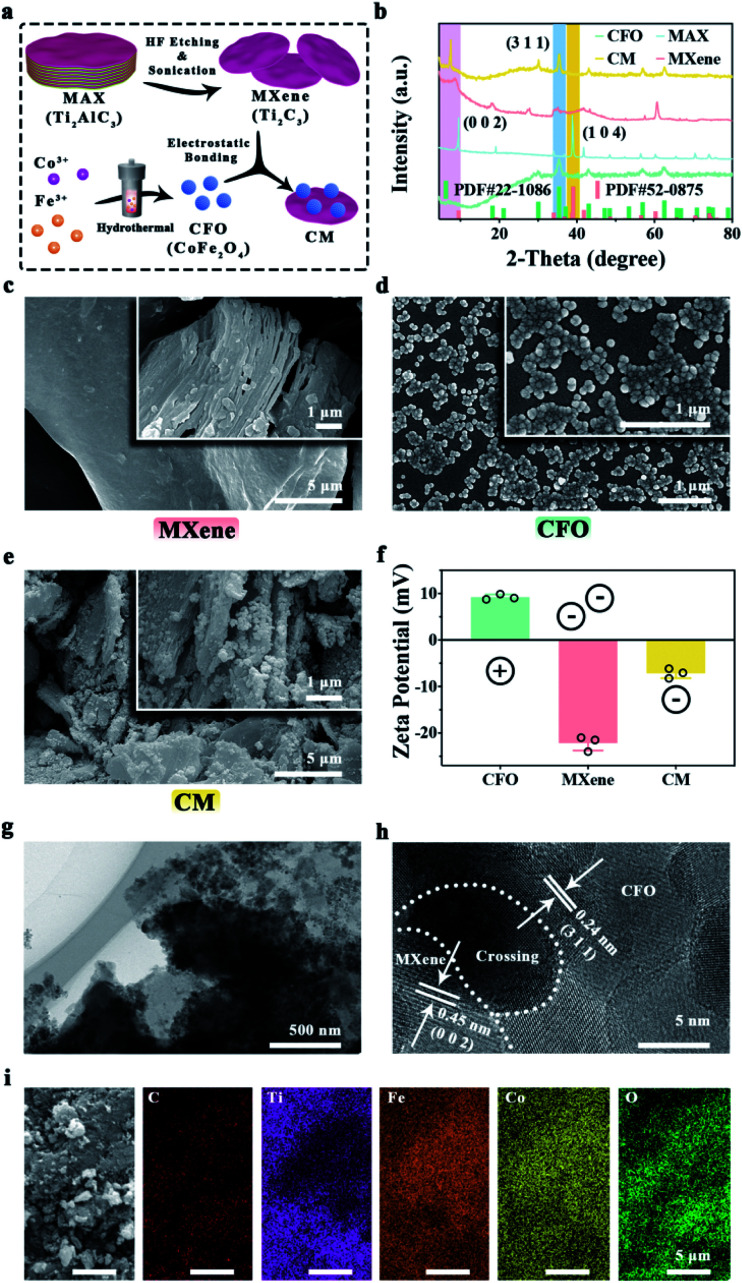
Characterizations of CFO, MXene, and CM: (a) illustration of the formation mechanism of CM nanoenzymes. (b) XRD patterns of CFO, MAX, MXene, and CM nanoenzymes. (c) SEM of multi-layer and single-layer MXene. SEM of CFO (d) and CM nanoenzymes (e). (f) Zeta potentials of CFO, MXene, and CM nanoenzymes. The low (g) and high (h) magnification TEM image of CM nanoenzymes. (i) EDS-mapping of CM.

To handle severe bacterial infection, we designed and developed novel nanoenzymes containing CFO and single-layer Ti_2_C_3_ MXene. As shown in Fig. 1a, CFO nanoparticles were synthesized by hydrothermal process, single-layer MXene was fabricated by *in situ* HF etch and ultrasonic exfoliate from Ti_2_AlC_3_ MAX, which can be combined by electrostatic bonding to form CM. XRD patterns (Fig. 1b) show that the characteristic peak of Ti_2_AlC_3_ MAX (PDF # 52-0875) at 39.0° is corresponded to (1 0 4) plane, which cannot be seen in Ti_2_C_3_ MXene, indicating the vanished Al layer. The peak which indicates (0 0 2) plane shifts from 9.5° to 9.0°, revealing the enlargement distance between Ti_2_C_3_ layers.^48^ SEM images (Fig. 1c) show the morphology of accordion-mimicking multilayer MXene and single-layer MXene. XRD patterns in Fig. 1b indicate the synthesis of CFO (PDF # 22-1086). Besides, the (3 1 1) plane of CoFe_2_O_4_ and (0 0 2) plane of MXene exist in the same XRD pattern, which explains the fabrication of CM nanoenzymes. SEM images of CFO (Fig. 1d) exhibit the uniform spherical nanoparticles. For CM nanoenzymes, there are obvious spherical CFO nanoparticles which adhere tightly on the single-layer MXene (Fig. 1e). To investigate the combination principle, zeta potentials of CFO, MXene and CM were detected. As Fig. 1f shows, CFO possesses a positive zeta potential (9.2 mV), and MXene shows an inverse negative zeta potential (−22.1 mV). After combination, the zeta potential of CM nanoenzymes raises to −7.1 mV because of the electrostatic interaction. The low-magnification TEM image of CM (Fig. 1g) shows that spherical CFO nanoparticles deposited on the surface of MXene. The high-magnification TEM image (Fig. 1h) shows the lattice spacing of CFO nanoparticles are approximately 0.24 nm, which is corresponded to its (3 1 1) planes. The lattice spacing of 0.45 nm can be attributed to the MXene interplanar spacing.^49^ The elemental mapping images of CM nanoenzymes demonstrate that C, Ti, Fe, Co, and O elements are present in the nanoenzymes (Fig. 1i). These results indicate the successful synthesis of CM nanoenzymes.

### 3.2. Photothermal and photodynamic performance

**Fig. 2:**
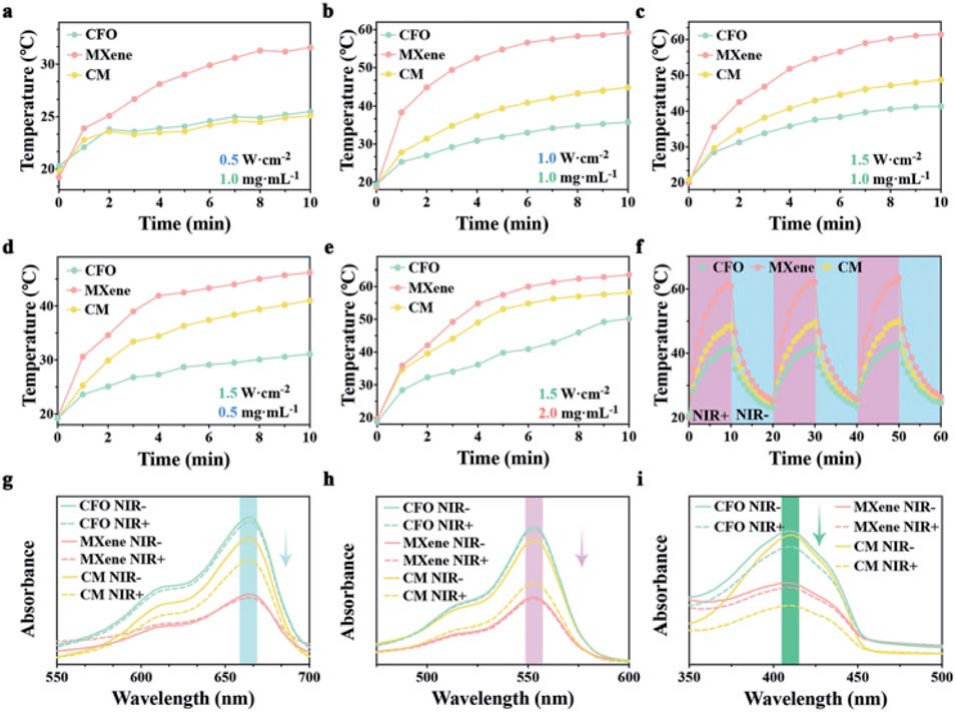
Photothermal and photodynamic properties detection: photothermal heating curve of CFO, MXene, and CM nanoenzymes (1 mg mL^−1^) under 0.5 (a), 1.0 (b), and 1.5 (c) W cm^−2^ NIR irradiation. (d and e) Temperature profiles of CFO, MXene, and CM nanoenzymes 0.5 and 2.0 mg mL^−1^ under 1.5 W cm^−2^ NIR irradiation. (f) The photothermal periodic curve of CFO, MXene, and CM nanoenzymes. UV-vis absorption changes of MB (g), RhB (h), and DPBF (i) with different samples.

CFO and MXene possess prominent photothermal ability, there, the effect of NIR power intensity on the photothermal properties were tested, the samples (1 mg mL^−1^) was exposed to laser with powers intensity of 0.5, 1.0, and 1.5 W cm^−2^, respectively. As shown in Fig. 2a, the samples with 0.5 W cm^−2^ exhibit the lower temperature (<35 °C). With power density of 1.0 W cm^−2^ (Fig. 2b), the maximum temperature of CM reaches 45 °C. After enhancing the intensity to 1.5 W cm^−2^ (Fig. 2c), the temperature of CFO, MXene and CM nanoenzymes can reach to 41, 61, and 49 °C, respectively. Thus, the maximum temperature would raise up with the increased laser power density. Moreover, the influence of samples concentration to photothermal properties was explored. Samples with other concentrations of 0.5 and 2.0 mg mL^−1^ (Fig. 2d and e respectively) were separately irradiated with a 1.5 W cm^−2^ NIR light. Combined with Fig. 2c, the temperatures of CM with 0.5, 1.0, and 2.0 mg mL^−1^ can rise from 20 to 41, 49, and 58 °C, respectively. Thus, it is obviously revealed that the rise of concentration of the samples will increase the maximum temperature. Taking these phenomena into consideration, the final temperature can be controlled by changing the intensity of 808 nm NIR light and the concentration of material solution to achieve efficient antibacterial performance and protect healthy tissue from the damage of high temperature simultaneously. Subsequent antibacterial experiments are conducted with 1.0 mg mL^−1^ of samples and 1.5 W cm^−2^ NIR light. The periodic temperature of CFO, MXene, and CM were documented with 1.5 W cm^−2^ NIR light for 10 min for verify the photothermal stability of the samples. Fig. 2f displays that no temperature variety is found after three cycles, which indicates that the CFO, MXene, and CM nanoenzymes present remarkable photothermal stability.

MB was utilized to detect the ROS production because it can be degraded by ˙OH.^50^ As shown in Fig. 2g, the absorption peaks at 664 nm of the CFO and MXene groups show no apparent changes, demonstrating that CFO and MXene lack the ability to generate ˙OH. The MXene groups displays the lowest peak, which may be attributed to the large specific surface of MXene that can adsorb MB. The absorption peak of the CM nanoenzymes with NIR light irradiation shows a noticeable decrease, revealing that CM nanoenzymes possess the remarkable capacity of generating ˙OH. RhB was also used as the ˙OH trapping agent to detect the photodynamic activity of samples.^51^ As shown in Fig. 2h, CM with the NIR light irradiation exhibits a sharp decrease, indicating that CM possesses superior photodynamic activity with the irradiation of NIR light, which is consistent with the MB test. DPBF can react with O_2_^−^ to reduce the original absorption peak at 415 nm,^52^ as an O_2_^−^ trapping agent. As shown in Fig. 2i, CFO and MXene display negligible changes after irradiating with NIR light, while the absorption intensity of CM with and without NIR light exhibit significant difference because the CM nanoenzymes generate O_2_^−^ by the surrounding oxygen captured the separated electrons. Thus, we make a conclusion that CM possesses superb photothermal performance and photodynamic activity.

### 3.3. Antibacterial effect

**Fig. 3:**
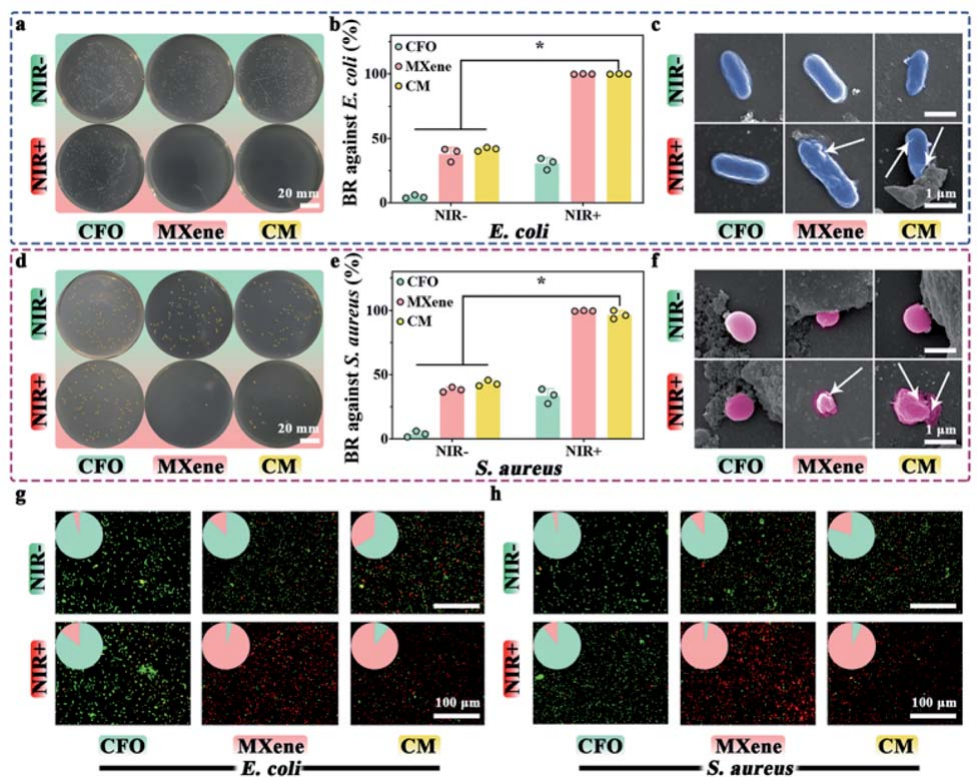
Antibacterial capability tests: spread plates of *E. coli* (a) treated with CFO, MXene, and CM nanoenzymes and the corresponding bactericidal rate (b). (c) SEM images of *E. coli* cultured with CFO, MXene, and CM and white arrows indicate the damaged bacterial membrane and the leakage of the bacterial containing. Spread plates of *S. aureus* (d) treated with CFO, MXene, and CM nanoenzymes and the corresponding bactericidal rate (e). (f) SEM images of *S. aureus* cultured with CFO, MXene, and CM and white arrows indicate the damaged bacterial membrane and the leakage of the bacterial containing. Fluorescent images of *E. coli* (g) and *S. aureus* (h) with live/dead staining. *p < 0.05.

Spread plate method was utilized to explore the antibacterial effect of materials for *E. coli* and *S. aureus*. As shown in Fig. 3a and d, none of the samples exhibits attractive antimicrobial properties in dark control groups. MXene and CM show the favorable antibacterial ability with *E. coli* and *S. aureus* after NIR light irradiation. Fig. 3b and e shows the BR of samples, it can be seen that BR of CFO against *E. coli* and *S. aureus* without NIR are approximately 4.7% and 3.7%, respectively. After being decorated with MXene, the BR rise to 41% and 43% respectively, indicating the antibacterial activity of MXene. In striking comparison, the BR of all samples have been enhanced upon the NIR light irradiation. Especially the BR of MXene and CM has increased to nearly 100%. These results demonstrate that MXene and CM with NIR irradiation have enhanced bactericidal ability.

The SEM morphology of *E. coli* (Fig. 3c) and *S. aureus* (Fig. 3f) with and without NIR light irradiation was scrutinized. Without NIR, *E. coli* appears as a capsule, while *S. aureus* appears as a smooth and irregular sphere. With NIR light irradiation, the bacteria with CFO still have an intact smooth membrane. In contrast, the morphology and structure of bacteria with MXene and CM are wrecked, which shows noticeable shrinkage, deformation, and cytoplasmic leakage (indicated by white arrows). It demonstrates that CM with NIR irradiation has an excellent inhibitory effect on the growth of surface pathogens, which is consistent with the results of the plate method.

The antibacterial property of different samples was further investigated with live/dead activity assays, live bacteria with intact membranes are in green fluoresce, whereas dead bacteria is red. The corresponding fluorescent images of *E. coli* and *S. aureus* are depicted in Fig. 3g and h. In the absence of NIR, all samples exhibit green (live bacteria), indicating suboptimal antibacterial properties. Intriguingly, The CFO samples were dyed green with or without NIR light treatment, which accords with the results of spread plate. For MXene and CM, more dead bacteria can be found after NIR irradiation, which is consistent with that in Fig. 3a and d, demonstrating their fantastic antibacterial potential against *E. coli* and *S. aureus*.

### 3.4. Antibacterial mechanism

**Fig. 4:**
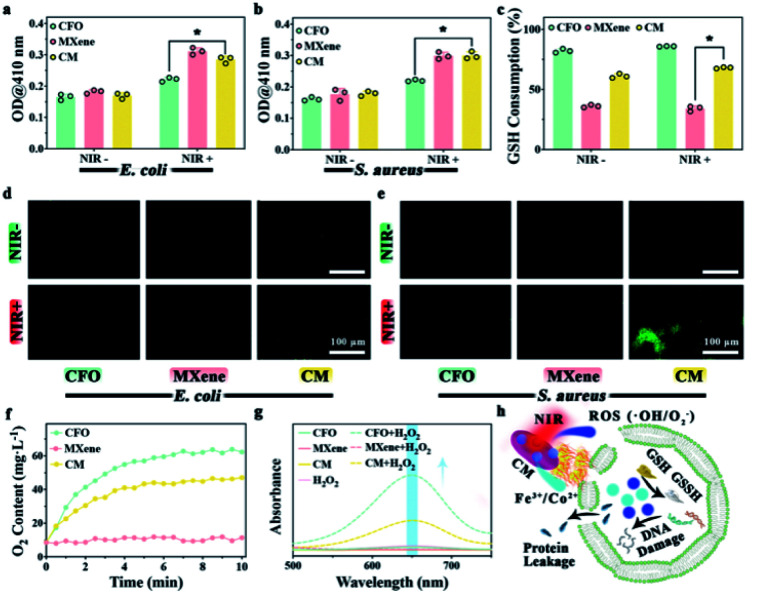
Antibacterial mechanism evaluations: permeability test of the bacterial membrane of *E. coli* (a) and *S. aureus* (b) by ONPG assay. (c) GSH consumption treated with CFO, MXene, and CM nanoenzymes. Fluorescence of intra-bacterial ROS induced by different samples in *E. coli* (d) and *S. aureus* (e). (f) O_2_ generation profile of different samples with H_2_O_2_. (g) UV-vis absorption changes of TMB with different samples with H_2_O_2_ present. (h) Scheme of the bactericidal mechanism. *p < 0.05.

To understand the antibacterial mechanism of CM nanoenzymes, the change of bacterial membrane permeation, GSH consumption, ROS level, and the enzymes-mimicking activity were investigated. Fig. 4a and b show the bacterial membrane permeation, all groups kept in the dark show no apparent OD values variation. In contrast, CM groups display a significantly higher OD values under NIR light irradiation, suggesting that phototherapy has a particular effect on the membrane permeation. Obviously, the MXene group compared to other groups exhibits higher permeability. The excellent photothermal effect of MXene indicates that local high temperature plays a vital role in enhancing the permeability of the bacterial membrane. Fig. 4c shows the GSH consumption rate after cultivation with different samples. There is no significant change in all groups whether NIR light is applied, indicating the Fe^3+^ released from CM is oxidized by GSH as eqn (5).

5 Fe^3+^ + GSH → Fe^2+^ + GSSH

H_2_O_2_ is continuously generated in the bacterial metabolism. To maintain a dynamic redox equilibrium, the self-defense mechanism will clear H_2_O_2_. Stress due to oxidation will produce denatured protein and nucleic acid when the balance is disrupted by increased ROS levels. Therefore, after proving samples *in vitro*, the ROS in bacteria was detected using DCFH-DA. As exhibited in Fig. 4d and e, the less fluorescence of CFO and MXene groups in both *E. coli* and *S. aureus* indicate little endogenous ROS in the bacteria. Compared to CM group without NIR light irradiation, the CM group with NIR irradiation show a more vigorous fluorescence intensity, illustrating that phototherapy promotes CM nanoenzymes to increase ROS levels in *E. coli* and *S. aureus*. O_2_ levels were tested in H_2_O_2_ solution incubated with each of the three samples shown in Fig. 4f. Compared to the MXene group, the other two groups show a stronger catalytic ability to convert H_2_O_2_ into O_2_, indicating that Fe^3+^ in samples can catalyze the H_2_O_2_ and produce O_2_ with CAT-mimicking property as eqn (6).

6 Fe^3+^ + H_2_O_2_ → Fe^2+^ + O_2_ + 2H^+^

In this section, the POD-mimicking activity was evaluated using TMB as a substrate. As shown in the Fig. 4g, CFO and CM groups in the presence of H_2_O_2_ show absorption peaks around 650 nm, suggesting that the Co^2+^ and Fe^3+^ in CM can catalyze the H_2_O_2_ to generate ˙OH through Fenton and Fenton-like reaction as eqn (7) and (8).

7 Co^2+^ + H_2_O_2_ → Co^3+^ + ˙OH + OH^−^

8 Fe^2+^ + H_2_O_2_ → Fe^3+^ + ˙OH + OH^−^

By combining the outcome of the experiment above, a possible antibacterial mechanism for phototherapy performance of CM nanoenzymes is exhibited in Fig. 4h. The CM nanoenzymes under NIR light irradiation will increase the permeability of the bacterial membrane to induce the leakage of intracellular proteins. Meanwhile, a mass of ROS and metal ions oxidize GSH to GSSH, which disintegrates the bacterial antioxidant defense system.^53^ Furthermore, the CM can act as a POD-mimicking catalyst to create endogenous ROS through the Fenton and Fenton-like reactions, which will further consume bacterial GSH.^54^ This process will finally elicit a high level of ROS within the bacteria and subsequently damage the intracellular DNA. These experimental results interpret the antibacterial mechanism of CM nanoenzymes.

### 3.5. Hemocompatibility assessment

**Fig. 5:**
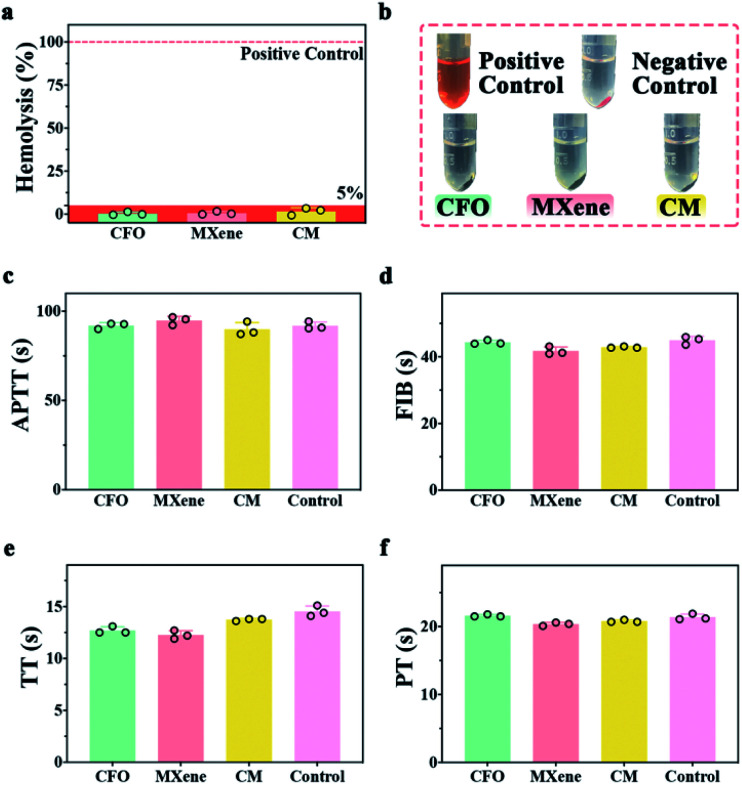
The hemocompatibility test of samples: (a) the hemolytic rate of CFO, MXene, and CM nanoenzymes. (b) Corresponding photographs of the samples in the hemolysis experiments. The results of APTT (c), FIB (d), TT (e), and PT (f) of the samples, respectively.

Since nanocomposites will contact blood for *in vivo* disease treatment, it is essential to detect the hemocompatibility of nanocomposites. Fig. 5a shows the hemolysis of CFO, MXene and CM, the hemolysis rates are lower than 5% when incubated with 2% whole blood at 37 °C for 3 h. The digital images (Fig. 5b) demonstrate that no heme escaped from the red blood cells in the supernatant derived from the hemolysis test. Blood coagulation assay was also investigated including APTT, FIB, TT, and PT.^55^Fig. 5c–f demonstrate that samples have no influence on blood coagulation when pure goat plasma is chosen as control. The result of hemolysis and blood coagulation assay illustrate the excellent hemocompatibility of CM.

### 3.6. *In vitro* and *in vivo* evaluation

**Fig. 6:**
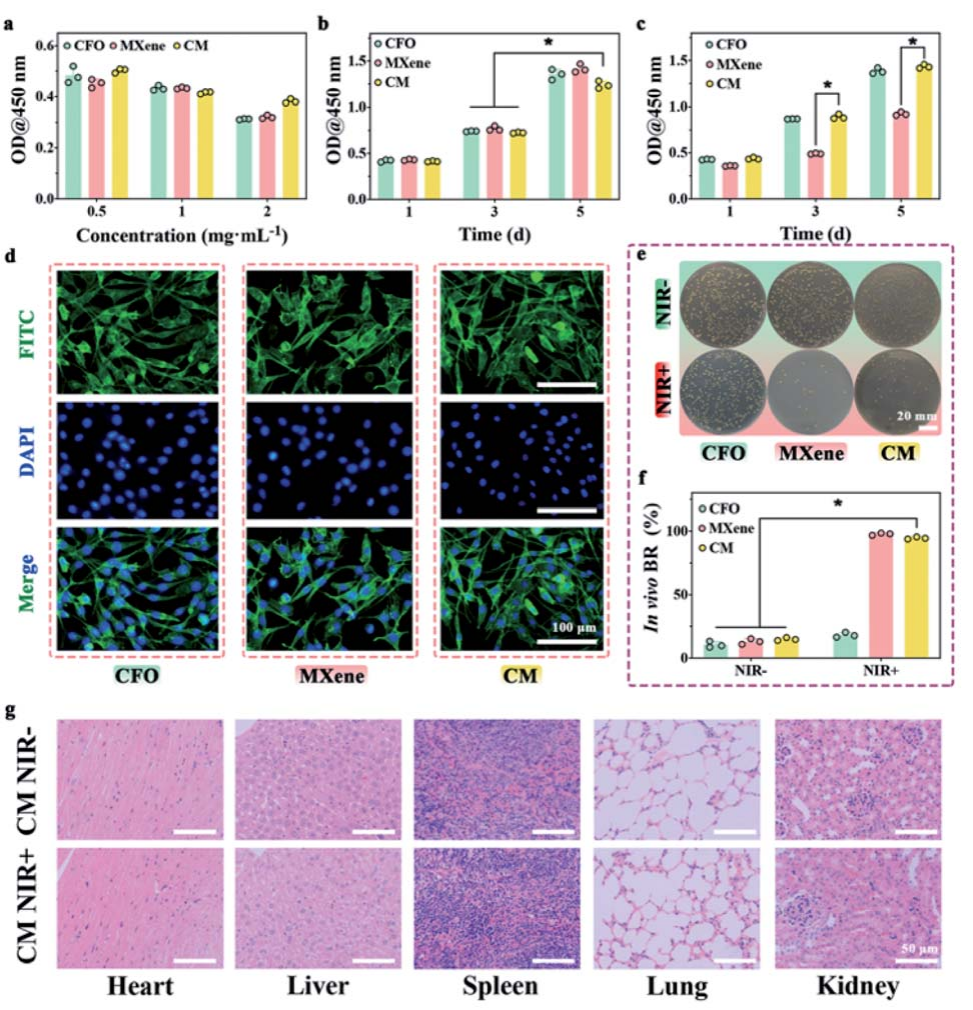
L929 *in vitro* and *in vivo* test: (a) viability of cells in different concentrations of samples for 1 d. (b) Viability of cells with varying samples for 1, 3, and 5 d. (c) Viability of cells with varying samples and 10 min NIR for 1, 3, and 5 d. (d) FITC/DAPI staining of cells cultured with samples. *In vivo* antibacterial test of *S. aureus* dispersed on the plate (e) and relevant BR (f). (g) H&E staining of heart, liver, spleen, lung, and kidney in CM NIR^−^ and CM NIR^+^ groups.

The cytotoxicity was evaluated by the CCK-8 test towards L929 cells. The activity of L929 cells gradually decreases as the concentration of the material increase (0.5, 1, and 2 mg mL^−1^), as shown in Fig. 6a. In other words, all samples exhibit roughly concentration-dependent cytotoxicity towards L929 cells. Here, we chose the concentration of 1 mg mL^−1^ for subsequent experiments. Fig. 6b displays that the OD values of L929 cells treated with CFO, MXene, and CM have an increased trend. The OD value at day 5 is almost 3 times that of the cells at day 1. With 10 min NIR irradiation, the cell viability of MXene group showed obvious decline in comparison with other groups, because of the local high temperature generated by MXene within the NIR irradiation. The excessive photothermal activity of materials could induce irreversible damage to cells and inhibit cell viability. FITC/DAPI staining (Fig. 6d) further confirm this result, it can be easily observed that after co-incubation for 3 d, the L929 cells incubated with all the samples exhibited a complete spindle-shaped cell morphology, indicating all samples have no cytotoxicity for cell proliferation.

The antibacterial, biocompatibility and biosafety were impact in a mouse skin wound model infected with *S. aureus* at the back of mice because of the remarkable antibacterial ability and cytocompatibility *in vitro*. The results of *in vivo* antibacterial evaluation (Fig. 6e and f) verify the extraordinary antibacterial ability of CM nanoenzymes. Furthermore, images of main organs (heart, liver, spleen, lung, and kidney) stained with H&E show that there are few side effects following phototherapy, which could be related to the biocompatibility and biosafety of CM (Fig. 6g).^56^ The results confirmed that CM nanoenzymes could keep a balance of antibacterial activity and biocompatibility with NIR *in vitro* and *in vivo*.

## 4. Conclusion

In summary, we succeeded in synthesizing new CM nanoenzymes compounded by CoFe_2_O_4_ and MXene for drug-free antibacterial agents. Compared with the previous drug-free agents, CM nanoenzymes exhibited the multi-enzyme-mimicking activities to generate ROS: (1) POD-mimicking activity to generate a considerable amount of ˙OH from H_2_O_2_ by Fenton and Fenton-like reaction of Fe(iii) and Co(ii); (2) GSHOx-mimicking property to consume interbacterial GSH in the redox cycling. In addition, CM nanoenzymes exhibit phototherapy under the NIR irradiation by photothermal and photodynamic processes. The CAT-mimicking activity of Fe(iii) produces O_2_ from H_2_O_2_ to enhance the PDT. The multi-enzyme-mimicking catalytic therapy and phototherapy synergistically improved bactericidal efficacy *in vitro* and *in vivo*. As biomedical materials, it is also highly biocompatible *in vitro* and *in vivo*. Overall, this research establishes a strong foundation for employing nanoenzymes to effectively regenerate bacteria-infested situations.

## Author contributions

Jiacheng Shi: conceptualization, methodology, formal analysis, investigation, writing – original draft. Rui Shu: supervision, funding acquisition. Xiuyuan Shi: writing – review & editing. Yunfei Li: writing – review & editing. Jiangge Li: methodology, formal analysis. Methodology, writing – original draft. Yi Deng: writing – review & editing, conceptualization, supervision, funding acquisition. Weizhong Yang: conceptualization, supervision, funding acquisition.

## Conflicts of interest

There are no conflicts to declare.

## Supplementary Material
